# Temperature-Driven Structural and Morphological Evolution of Zinc Oxide Nano-Coalesced Microstructures and Its Defect-Related Photoluminescence Properties

**DOI:** 10.3390/ma9040300

**Published:** 2016-04-20

**Authors:** Karkeng Lim, Muhammad Azmi Abdul Hamid, Roslinda Shamsudin, N.H. Al-Hardan, Ishak Mansor, Weesiong Chiu

**Affiliations:** 1 Materials Science Program, School of Applied Physics, Faculty of Science and Technology, Universiti Kebangsaan Malaysia, Selangor Darul Ehsan 43600, Malaysia; linda@ukm.edu.my (R.S.); n.h.alhardan@gmail.com (N.H.A.-H.); 2 Technical Support Division, Malaysian Nuclear Agency, Kajang 43000, Malaysia; ishak_mansor@nm.gov.my; 3 Low Dimensional Materials Research Centre, Department of Physics, Faculty of Science, University of Malaya, Kuala Lumpur 50603, Malaysia; w.s.chiu@um.edu.my

**Keywords:** zinc oxide, thin films, vapor deposition, photoluminescence spectrofluorometer, X-ray photoelectron spectroscopy, surface structure defects

## Abstract

In this paper, we address the synthesis of nano-coalesced microstructured zinc oxide thin films via a simple thermal evaporation process. The role of synthesis temperature on the structural, morphological, and optical properties of the prepared zinc oxide samples was deeply investigated. The obtained photoluminescence and X-ray photoelectron spectroscopy outcomes will be used to discuss the surface structure defects of the prepared samples. The results indicated that the prepared samples are polycrystalline in nature, and the sample prepared at 700 °C revealed a tremendously *c*-axis oriented zinc oxide. The temperature-driven morphological evolution of the zinc oxide nano-coalesced microstructures was perceived, resulting in transformation of quasi-mountain chain-like to pyramidal textured zinc oxide with increasing the synthesis temperature. The results also impart that the sample prepared at 500 °C shows a higher percentage of the zinc interstitial and oxygen vacancies. Furthermore, the intensity of the photoluminescence emission in the ultraviolet region was enhanced as the heating temperature increased from 500 °C to 700 °C. Lastly, the growth mechanism of the zinc oxide nano-coalesced microstructures is discussed according to the reaction conditions.

## 1. Introduction

Zinc oxide (ZnO) has been widely studied and has received noteworthy attention since 1935 [1], as our daily lives and current industries crucially rely on this compound. It has been recognized as one of the well-known potential materials that could be beneficial and serviceable for electronic and optoelectronic applications owing to its attractive properties. For instance, ZnO has a direct and wide band gap energy of 3.37 eV, which enables it to be transparent in visible light and to operate in the ultraviolet (UV) and blue wavelength regions [2]. Moreover, ZnO has a large exciton binding energy of approximately 60 meV that ensures an efficient excitonic emission at room temperature, enabling it to be suitable for UV optoelectronic applications [3]. This notable exciton binding energy of ZnO makes it a more attractive material than other compounds, especially GaN, a compound with exciton binding energy of 26 meV. A number of methods have been reported in the literature regarding the synthesis of ZnO materials with a range of different morphologies, such as thermal evaporation [4,5], carbothermal reduction [6], magnetron sputtering [7], cathodic electrodeposition [8,9], hydrothermal synthesis [10,11], and sol-gel processing [12,13]. However, the thermal evaporation approach remains as a valuable technique and can be interpreted as a cost effectiveness technology for the growth of metal oxide micro- or nanostructures. There is little research reporting the achievability of growing ZnO at comparatively low temperature utilizing the liquid phase growth methods such as hydrothermal synthesis and sol-gel processing. However, there are some drawbacks relating to these processes in contrast to the thermal evaporation method, such as: (i) these methods demanded additional fabrication step, for example furnishing a seed layer by physical methods prior to the actual synthesis [14]; (ii) these methods required a precise control of the composition, pH, and also concentration of an electrolyte solution; (iii) the growth rate of these methods was inferior to the vapor phase process [15]; and (iv) these methods needed a post thermal annealing treatment to alter the thin film’s internal crystallization and concurrently reduced the defects in the thin film and then prevented the growth of other crystalline phases [16].

Miscellaneous factors have been defined from the thermal evaporation method that leads to the formation of ZnO with contrastive morphologies and properties. However, not all the factors are of requisite. Temperature is the key parameter, which ought to be contemplated for the formation of diverse ZnO morphologies before employing it in various applications. Several research groups have deliberated the dependency of temperature and luminescence properties of ZnO [17,18,19]. However, they have added graphite powder as a catalyst in their experiments and run at source temperature up to the 950 °C. In particular, the presence of a catalyst in the fabrication is not only detrimental to the intrinsic properties of the materials but also deleterious to its final product applications due to the existing contamination. Using an extreme temperature to locally heat the reactant source in order to enable the vaporization will restrict the fabrication of ZnO micro and nanomaterials in gentle circumstances [20,21]. Furthermore, Lee [22] reported that luminescent property, morphology, and microstructure of ZnO nanostructures are related to the oxidation rate of Zn film. Very recently, Yuan *et al.* [23] studied the dependence of different oxidation temperatures on the growth morphologies and microstructure of ZnO. However, they did not study the luminescence properties.

The photoluminescence (PL) of ZnO have been comprehensively studied. It is the spontaneous emission of light from a material following an optical emission. The motivation of PL studies is to elucidate the correlative between the origins of different emission bands to the origins of different ZnO defect emissions [24]. Typically, ZnO exhibits a near band edge UV emission and a broad defect-related visible emission. This visible emission is often described in green luminescence, although violet, blue, yellow, and orange-red luminescence have also been reported [25,26]. Nevertheless, the understanding of ZnO defects by PL principle is still inadequate and remains controversial.

Here, we report the synthesis of ZnO nano-coalesced microstructures with varied morphologies via thermal evaporation without any metal catalyst or pre-deposited ZnO seed layer at 500 °C, 600 °C, and 700 °C, respectively. We comprehensively studied the connection between the surface structure defects with PL and XPS spectra as other researchers did not focus on this. Under this circumstance, results from the XPS spectra will be used to support the PL analysis so that a better prediction and dissection regarding to the origins of surface structure defects can be derived in this study. The inherent factors primarily influencing the material properties and growth mechanism will be explored as well.

## 2. Results and Discussion

**Figure 1:**
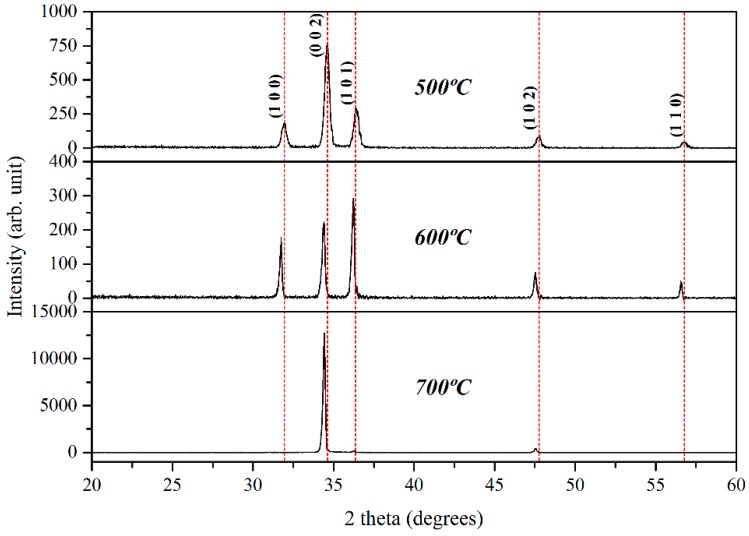
XRD patterns of as-deposited ZnO prepared at different growth temperatures.

The XRD patterns of the deposited ZnO samples on SiO_2_-coated Si substrates at different growth temperatures are illustrated in Figure 1.

The XRD diffraction peaks can be assigned to (100), (002), (101), (102), and (110) hexagonal wurtzite structures of ZnO, with the cell parameter of *a* = *b* = 3.242 Å and *c* = 5.188 Å according to the JCPDS card no. 01-079-0205. The diffraction peak at a Bragg angle of around 34.5°, corresponding to the (002) plane overwhelmingly reveals the preferred orientation of ZnO growth at 500 °C and also 700 °C. This close-packed plane is predictably dominant for ZnO, as it has the lowest surface energy among the possible orientations of hexagonal wurtzite ZnO [22,27]. However, ZnO grown at 600 °C has a preferred orientation along the (101) plane, compared to the ones on 500 °C and 700 °C. This unfavorable crystal plane appears to be related to the oxidation rate, which leads to inclusion of the absorbed oxygen atoms in the ZnO lattice [22]. Thus, with the increase in oxidation temperature, the ZnO should present preferential growth in the (101) orientation. However, our XRD results are in contrast with this statement. The XRD peak intensity is related to many factors, which comprise the crystallization quality, density, and also thickness of thin films. Shifting of preferred orientation from the (002) to (101) plane for 600 °C sample is predominantly due to the dissimilarity of these factors. As shown in Figure 3, Figure 4 and Figure 5, numerous ZnO-branched nanowires (Figure 4) are formed on the substrate surface prepared at 600 °C compared to the quasi-mountain chain-like ZnO (Figure 3) and pyramidal textured ZnO (Figure 5) obtained at 500 °C and 700 °C, respectively. It is suggested that the strong (101) peak can be ascribed to the ZnO-branched nanowires formed with high density and also a different dimensional structure. The intensity of the (101) peak appears to reflect the density of ZnO-branched nanowires, which is identical as reported by other researchers [22,28]. No peaks for the unreacted metallic zinc and no other impurities were detected confirming the full conversion of the zinc metal to ZnO and the high purity of the obtained products.

**Figure 2:**
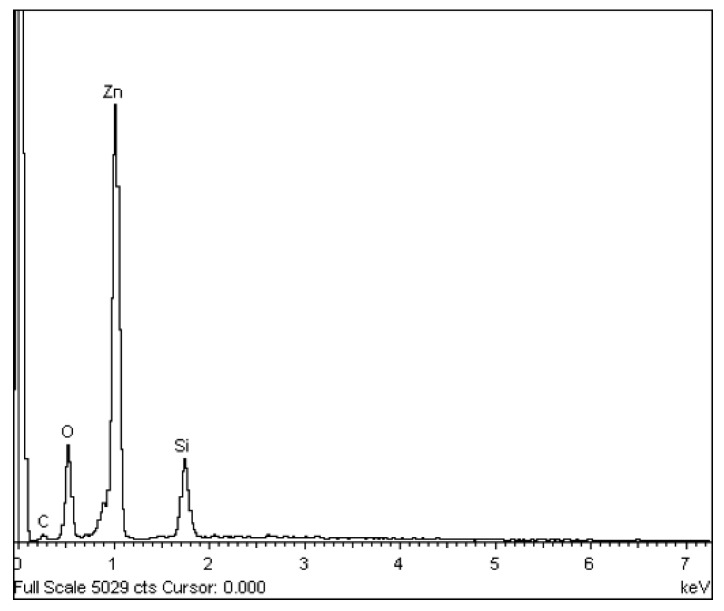
A typical EDX spectrum of as-synthesized ZnO at 500 °C.

A representative EDX spectrum presented in Figure 2 depicts a Si-related peak, which originated from the substrate, while the C peak is caused by the carbon contamination. Carbon deposits occur due to the interaction of the electron beam with the hydrocarbon molecule vapors that exist in the vacuum chamber or on the substrate surface [29]. Moreover, only zinc and oxygen atoms are seen in the EDX spectrum, proving the obtainment of pure ZnO products, which was also confirmed through XRD spectrum. The quantitative EDX analysis indicated that the oxygen content was intense than the zinc. However, the atomic composition ratio of oxygen to zinc could not be quantified, as a portion of the oxygen originated from the SiO_2_-coated Si substrate.

**Figure 3:**
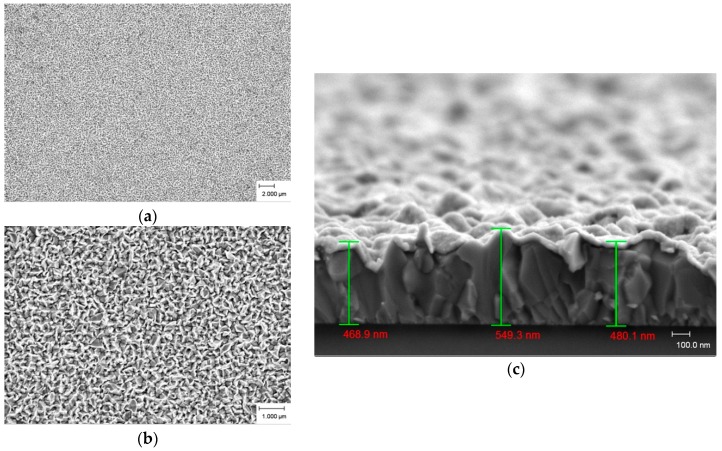
FESEM images of as-deposited ZnO products prepared at 500 °C. (**a**) low magnification; (**b**) high magnification; and (**c**) cross section.

**Figure 4:**
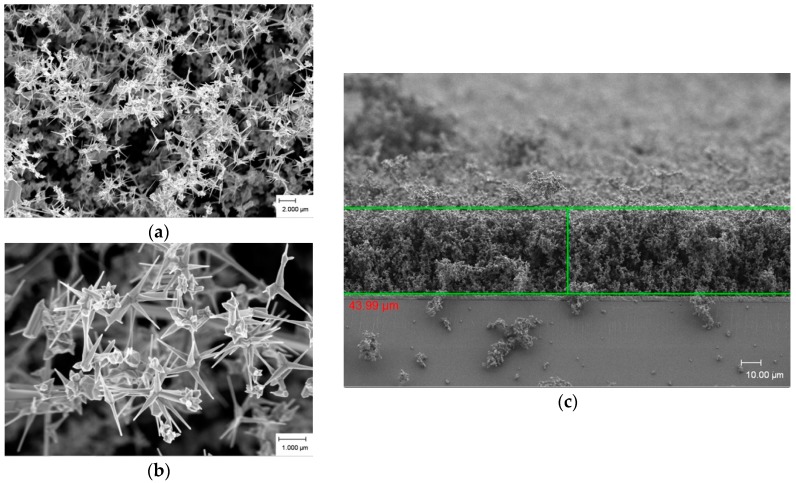
FESEM images of as-deposited ZnO products prepared at 600 °C. (**a**) low magnification; (**b**) high magnification; and (**c**) cross section.

**Figure 5:**
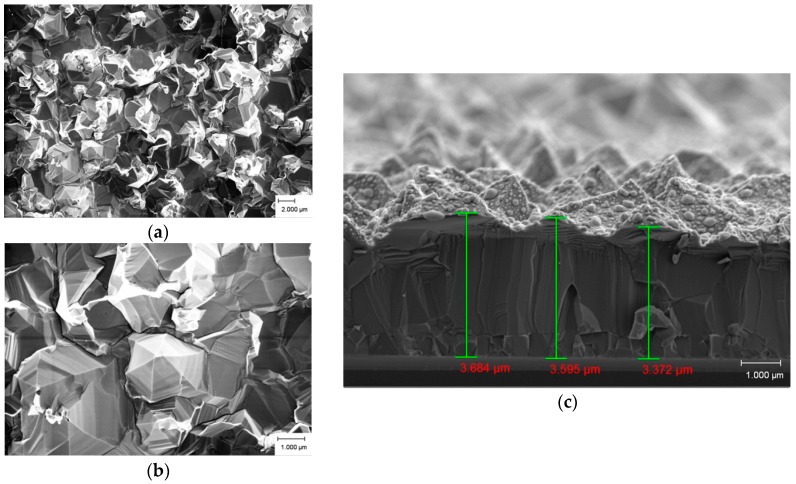
FESEM images of as-deposited ZnO products prepared at 700 °C. (**a**) low magnification; (**b**) high magnification; and (**c**) cross section.

The FESEM images of the ZnO as-deposited products with different growth temperatures in the plan view (low and high magnification) and cross section are presented in Figure 3, Figure 4 and Figure 5. Figure 3a,b show a dense, multi-directional, and irregular shaped quasi-mountain chain-like ZnO structure growth at 500 °C. Dimensions are difficult to measure as a consequence of these structures’ growth in 3D prism- and pyramid-like form with an irregular base. From the cross-section view (Figure 3c), we noticed a continuous powdery thin ZnO layer grown above a close-packed ZnO structure. The overall thickness of the quasi-mountain chain-like ZnO thin film ranges from 460 nm to 550 nm.

Figure 4a,b illustrate ZnO-branched nanowire growth from an agglomeration nucleus at 600 °C. These microstructures grew uniformly and decreased gradually like a needle at the end, with a diameter of about 40 nm to 250 nm and a length of more than 1 μm. From Figure 4c, we can clearly see some ZnO-branched hexagonal structures with sharp tip growth at the end of the branched nanowires. The thickness for ZnO-branched nanowire structure thin film is around 44 μm.

Figure 5a,b elucidate an irregular base of pyramidal textured ZnO structures obtained at 700 °C. Some of these structures display a small flat hexagonal facet at the top and some indicate sharp tips at the end. From the cross section view (Figure 5c), we observed that granular ZnO was deposited on the dense structure. The thickness of this thin film is in the range of 3.3 μm to 3.7 μm. It should be noted that the pyramidal ZnO textured structures obtained at 700 °C were densely packed, which results in the dominant diffraction peak of the (002) phase in the XRD results.

Growth temperature is a critical experimental parameter, which determines the kinetic energy for the formation of different morphologies of ZnO. It has an impact on the quantity of the reactive vapor to be generated and the surface diffusion length of the absorbed vapor types [30]. Temperature may facilitate various degrees of supersaturation of ZnO in its gaseous state, resulting in different structures [31]. At high temperature of 700 °C, more Zn or Zn suboxide vapors were produced to form a thick film. Whereas at a moderate temperature of 600 °C, the vaporization rate of Zn or Zn suboxides was lower than that at 700 °C. This means the exhaustion of gaseous product was slower at sintering temperature of 600 °C than at 700 °C. Hence, a thicker film was formed. In the case of 500 °C, only some Zn or Zn suboxides was formed, so that the thickness of film reduced dramatically. Vapor-solid (VS) and vapor-liquid-solid (VLS) processes are always used to describe the growth mechanism of ZnO micro and nanostructures. The VLS model is usually used to explain the growth mechanism at high temperatures, while the VS model dominates at low temperatures [32]. However, we have different views on this point. Based on our opinion, the VS model is more suitable and fulfills our sample growth mechanism since no “catalyst ball” is found in the tips of the structures but there is a reduction in dimensions of the samples.

**Figure 6:**
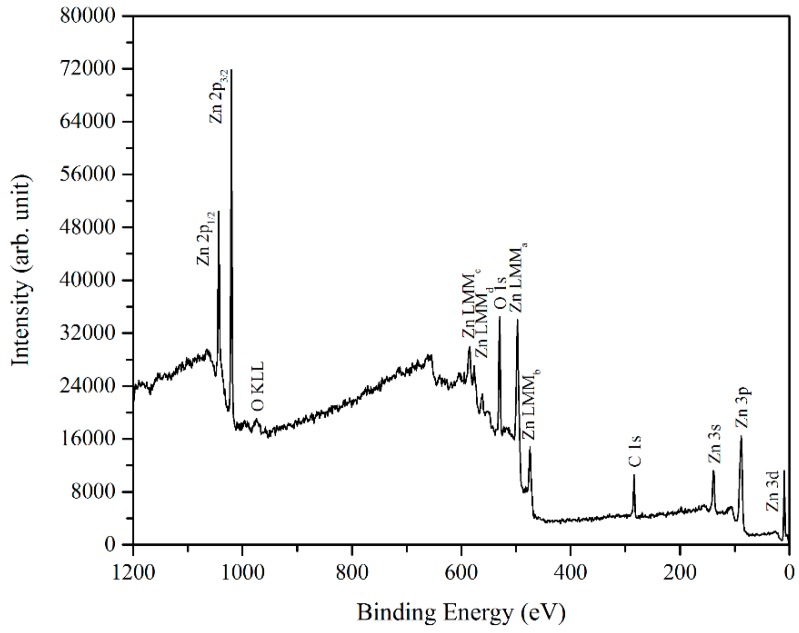
XPS wide scan of ZnO grown at 500 °C.

The chemical states of the prepared ZnO were investigated by the XPS measurements. The wide scan spectrum of the sample prepared at 500 °C is depicted in Figure 6. Here, the typical ZnO XPS spectrum can be seen. The mean peaks are carbon, oxygen, and Zn.

**Figure 7:**
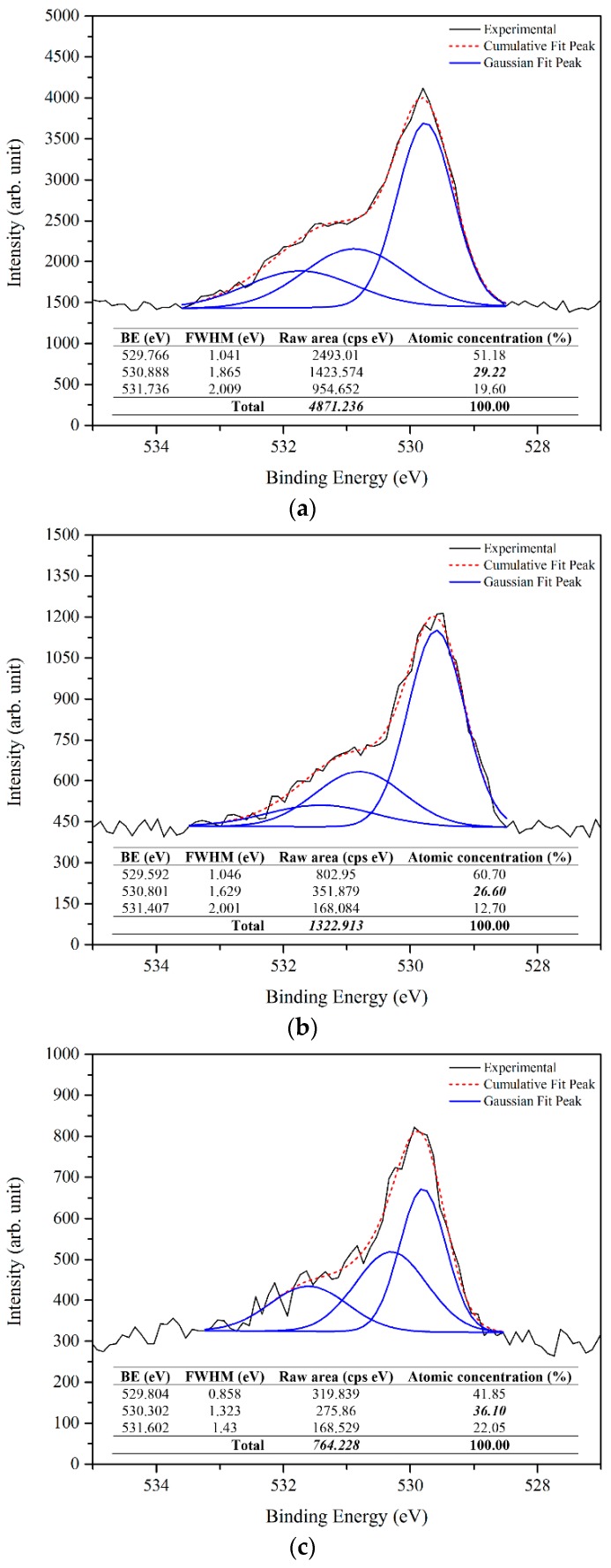
XPS analysis of oxygen 1s for as-deposited ZnO product grown at: (**a**) 500 °C; (**b**) 600 °C; and (**c**) 700 °C.

**Figure 8:**
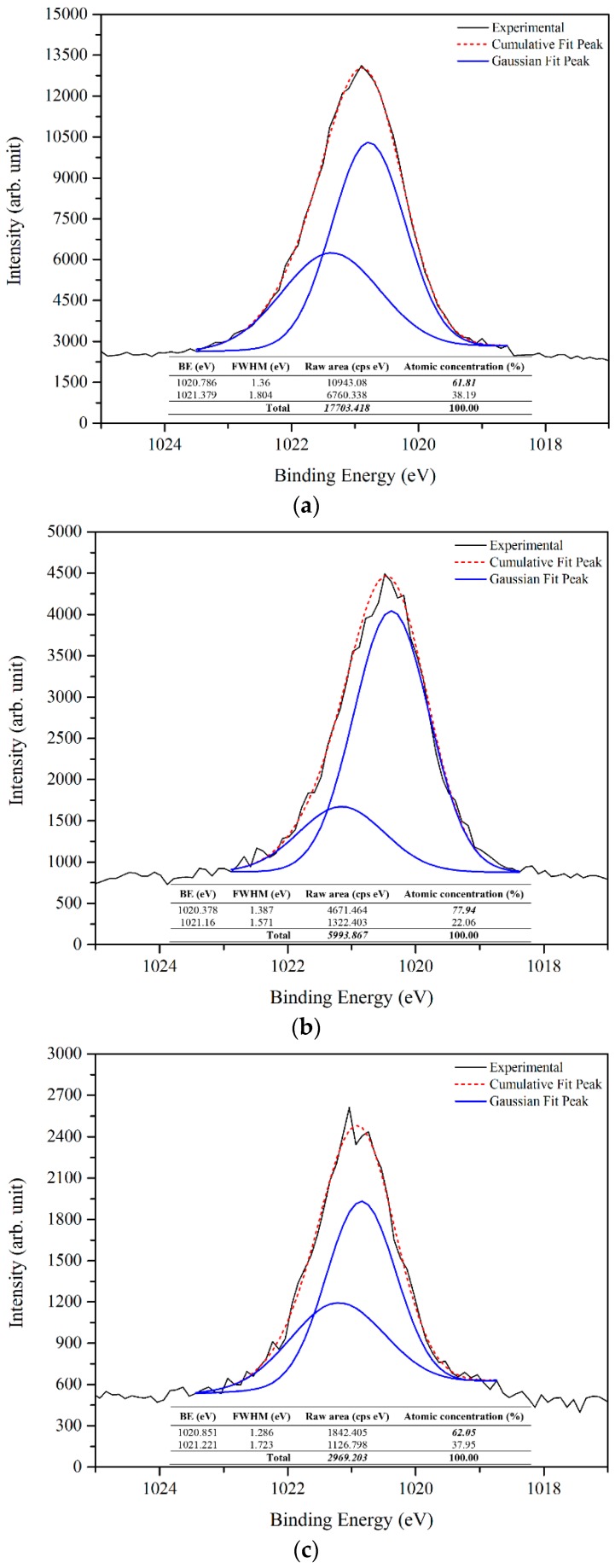
XPS analysis of zinc 2p_3/2_ for as-deposited ZnO product grown at: (**a**) 500 °C; (**b**) 600 °C; and (**c**) 700 °C.

The carbon peak at the binding energy of 284.5 eV originates from the sample surface because of its contamination, and is used for the internal calibration of the system [33]. For further information about the regions of interest above, a narrow and high-resolution scan was employed for the elements. Figure 7 and Figure 8 depicted the narrow scan of the oxygen 1s and Zn 2p_3/2_ for the samples prepared at 500 °C, 600 °C, and 700 °C, respectively.

The deconvoluted oxygen 1s XPS spectra (using the XPS peak fit software) of prepared samples shown in Figure 7 reveals the oxygen ions (O^2−^) in the wurtzite structure of hexagonal ZnO at 529 eV, whereas the peaks at 530 eV and 531 eV are attributed to the O^2−^ in oxygen-deficient regions (oxygen vacancies or defects) and O_2_ species (chemisorbed or dissociated oxygen), respectively [34,35,36,37]. The difference of each deconvoluted peak reveals the significant weight of each component of the oxygen 1s. Further, the Zn 2p_3/2_ peak at binding energy of 1021 eV can be deconvoluted to two components, the 1020 eV belonging to pure Zn (Zn interstitial) and the 1021 eV depicted to the Zn–O in the ZnO crystal [33,38,39]. Based on the rules of quantitative analysis of the XPS spectrum, the relative atomic surface concentrations of elements can be determined using the integrated area under principal peaks and a formula for quantification [33,40] as follows:

(1) $$C_{x}\;=\;\frac{I_{x}/S_{x}}{\sum_{i\;=1}I_{x}/S_{x}},$$

where *C_x_* is the atomic concentration of element *x*, *I_x_* is the integral intensity in Gaussian fitted XPS spectrum of element *x*, and *S_x_* is the relative sensitivity factor for element *x* based on the spectroscopy used. The relative atomic concentration of each component is shown in Table 1.

From the results, it can be deduced that the prepared ZnO contains Zn interstitial and oxygen vacancies, and the relative atomic concentration percentage of Zn interstitial and oxygen vacancies was the highest in the sample prepared at 500 °C. It is clear that at higher temperatures more reactions of Zn and O result.

**Figure 9:**
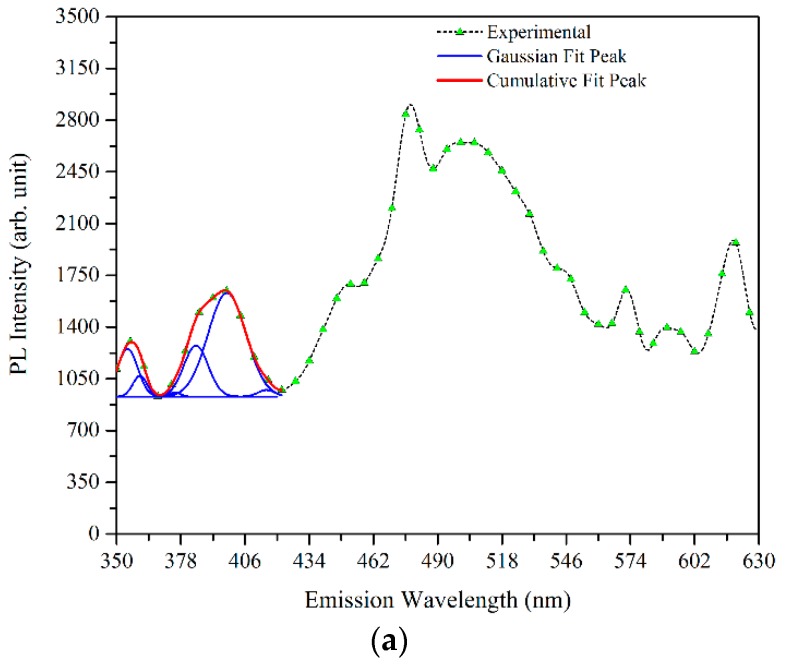

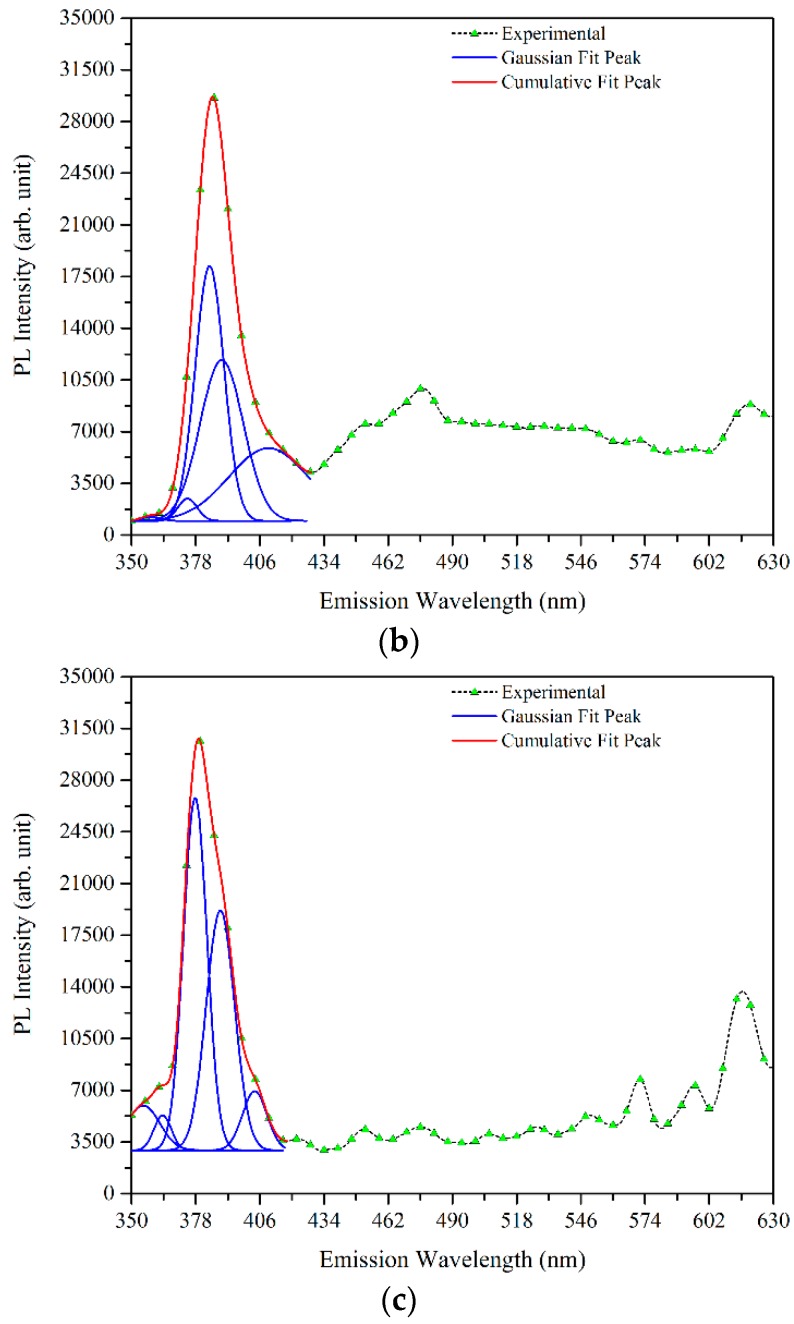
PL analyses of the prepared ZnO samples grown at: (**a**) 500 °C; (**b**) 600 °C; and (**c**) 700 °C.

Figure 9 presents the room temperature PL spectra filters by Schott glass filter with 330 nm cut-off wavelengths for the prepared ZnO at different growth temperatures.

The Gaussian deconvoluted spectra exhibit multi emission traps in the UV range from 350 nm to 370 nm and from 370 nm to 390 nm. These multi emission traps (blue lines) can be overlapped and combined to form a prominent peak (red dot line), which can be seen in Figure 9. The former range emission can be associated with the interband transition of the photogenerated electrons and holes. Relatively, a considerably stronger irradiation of excitation light will create a quasi Fermi level inside the conduction band. Therefore, the recombination of electron-hole pairs from this level to valence band will emit a photon energy that is greater than the ZnO energy band gap (3.37 eV) [41,42]. The latter range emission dovetails to the near band edge emission, which arises from the recombination of free excitons through an exciton-exciton collision process. The width of the UV emission indicates that the peak consists of sundry emissions. The emissions can be assigned to the two-phonon replicas due to the two transverse optical phonons within a range of separation from 50 meV to 110 meV [22]. The PL spectra also revealed a broad range of visible emission. Several defect-related recombination centers, such as Zn vacancy (*V*_Zn_), oxygen vacancy (*V*_O_), interstitial Zn (*Zn*_i_), interstitial oxygen (*O*_i_), Zn antisite (*Zn*_O_), and oxygen antisite (*O*_Zn_) defects were responsible for the luminescence process of ZnO in the visible region of electromagnetic radiation [43]. Among the defects, *Zn*_O_, is unlikely to be stabilized under equilibrium conditions ascribed to their high formation energies. Additionally, *Zn*_i_ and *V*_O_ can yield free electrons (donor type defects), whereas *V*_Zn_, *O*_i_, and *O*_Zn_ consume free electrons (acceptor type defects) in a ZnO crystal. The relative content of donors and acceptors determines the semiconductive property and further semiconductor-related properties of ZnO [44,45]. The observed PL spectra revealed emission band at 390 nm to 455 nm (violet luminescence) is probably attributed to the radiative defects related to the interface traps existing at the grain boundaries and emitted from the radiative transition between this level and the valence band [46]. The band from 455 nm to 492 nm (blue luminescence) is due to the single ionized *V*_Zn_^−^ [47], while the band from 492 nm to 577 nm (green luminescence) is commonly referred to as a deep-level or a trap-state emission attributed to the single ionized *V*_O_ and the emission results from the radiative recombination of a photogenerated hole with an electron occupying the *V*_O_ [48]. Furthermore, the band from 577 nm to 622 nm (yellow-orange luminescence) is attributed to the transition from the bottom of the conduction band to the *O*_i_ [49].

**Figure 10:**
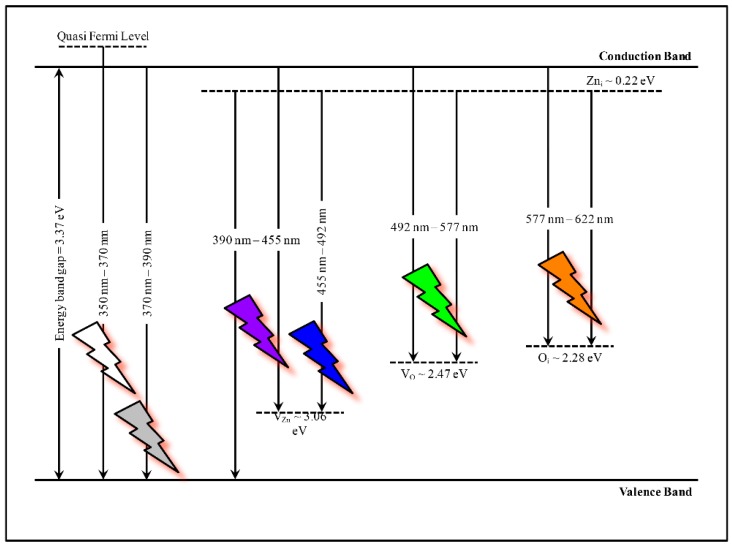
Schematic energy level diagram of various defect level emissions in ZnO.

Theoretically, the positions of these bands have been predicted. This can be explained by the full potential linear muffin-tin orbital method, which explains that the positions of the *Zn*_i_, *V*_Zn_, *V*_O_, and *O*_i_ are located at 0.22 eV, 3.06 eV, 2.47 eV, and 2.28 eV below the conduction band, respectively [50]. A detailed schematic energy level diagram of various level emissions in ZnO has been illustrated in Figure 10, which gives a vision for the recombination of electron-hole pairs from various defects back to the valence band and then emits photon energy, which fully supports our previous PL explanation.

The origin of the ZnO PL emission is a complicated process, as it is most related to the oxygen vacancies and/or Zn interstitial. Furthermore, it is a difficult task to correlate the PL emissions with optical transitions. Thus, PL emissions from ZnO defect energy levels are extremely complex and still not fully understood. However, the results from the XPS and PL support each other. From Figure 7 and Figure 8 it can be shown that the raw area of oxygen vacancies and Zn interstitial (bolded and italicized) from the respective total raw area decreases as the temperature increases. Also, the relative intensity of the UV near band edge emission to the visible emission from the PL results (Figure 9) increases as the temperature increases, which point to the improvement of ZnO quality. Consequently, the results indicated that the sample prepared at 500 °C has a higher percentage of oxygen vacancies and also Zn interstitial. In addition, the percentage of oxygen vacancies and Zn interstitial were enhanced as the temperature increased.

## 3. Materials and Methods

Cleaved single polished appearance SiO_2_-coated Si substrates with dimensions of 12 × 12 mm^2^ were used as the ZnO film growth medium. Prior to the growth, the substrates were ultrasonically cleaned sequentially with the acetone, methanol, and isopropyl alcohol, sonicated for 15 min in each respective solvent, and followed by a rinse with deionized water. The cleaned substrates were dried in a stream of air and underwent a dehydration bake in an oven at 100 °C to remove the trapped and absorbed moisture from the substrate surface. The growth process was carried out by the thermal evaporation method in a conventional commercial horizontal three-zone temperature alumina tube furnace. Metallic zinc powder (Emsure^®^ grade; particle size <45 nm; Merck KGAa, Darmstadt, Germany) of approximately 1.50 g was dispersed evenly in a rectangular alumina boat in the absence of any catalyst. The boat was loaded into the center of the tube of the horizontal furnace afterwards with the substrate located above the source at an estimated vertical distance of 15 mm with the single polished surface facing downward. As the temperature began to rise from room temperature with a program ramp rate of 10 °C/min, high purity argon gas was purged into the tube throughout the heating process with a flow rate of 800 sccm, providing a shield against environmental contamination. The flow of argon gas was stopped immediately once the temperature reached 419 °C (melting point of zinc), and the heating process was continuous done until it achieved the desired temperature of 500 °C, 600 °C, and 700 °C, respectively. A mixture of 1.6% oxygen and 98.4% argon as a reactant gas flowed throughout the tube maintaining the temperature for 30 min. The ZnO as-deposited product was allowed to cool inside the sealed tube furnace to room temperature naturally after a 15 min flow of argon gas in order to substitute the entire oxygen gas residue inside the tube.

The surface morphologies of the ZnO structures were examined using field emission scanning electron microscopy (FESEM, Carl Zeiss Supra 55VP, Jena, Germany) coupled an energy dispersive X-ray capability (EDX, Oxford Inca, Abingdon, UK) to quantify the elemental compositions. A glazing incidence X-ray diffractometer (XRD, Bruker D8 Advance, Karlsruhe, Germany) using copper-monochromatized Cu Kα1 radiation (λ = 0.154 nm) with an accelerating voltage of 40 kV and a current of 40 mA was used to determine the phase structures. The diffractograms were recorded in the 2θ range of 20° to 60° with a controlled step size of 0.025°. The surface compositions of the as-deposited products were analyzed by XPS (Kratos Axis Ultra DLD, Manchester, UK). Binding energies were corrected for the charge shift using the C 1s peak of carbon (*BE* = 284.5 eV) as a reference [37]. For the optical emission measurements, the PL spectra were recorded using a filter spectrofluorometer (Edinburgh Photonics, model FLSP 920, Livingston, UK) as an excitation source. The PL spectra were measured in the range of 350 nm to 630 nm with a 325 nm excitation wavelength at room temperature.

## 4. Conclusions

In summary, the nano-coalesced microstructured ZnO thin films were prepared via a thermal evaporation approach. The starting temperatures varied from 500 °C to 700 °C with the flow of oxygen and argon gas mixture. The results show enhancements on structural and optical properties of the contrastive ZnO morphologies. The near band edge UV emission of the prepared ZnO samples was enhanced with the temperature. Connection between the surface structure defects with PL and XPS spectra have been proven. Based on the obtained results, the growth mechanism of ZnO nano-coalesced microstructures was built up and discussed.

## Figures and Tables

**Table 1:** Relative atomic concentration for components in oxygen to zinc.

Sample	Component	Raw Area (cps eV)	Atomic Concentration (%)
500 °C	Oxygen	4871.236	58.18
	Zinc	25,094.375	41.82
**Total**		**29,965.611**	**100.00**
600 °C	Oxygen	1322.913	52.39
	Zinc	8614.431	47.61
**Total**		**9937.344**	**100.00**
700 °C	Oxygen	764.228	56.12
	Zinc	4281.111	43.88
**Total**		**5045.339**	**100.00**
